# Carburization of Tantalum Metal Powder Using Activated Carbon

**DOI:** 10.3390/ma18122710

**Published:** 2025-06-09

**Authors:** Seonmin Hwang, Dongwon Lee

**Affiliations:** Lightweight Materials Research Division, Korea Institute of Materials Science (KIMS), 797 Changwon-daero, Seongsan-gu, Changwon-si 51508, Gyeongsangnam-do, Republic of Korea; seonmin@kims.re.kr

**Keywords:** tantalum carbide, activated carbon, carburization, tantalum, graphite, carbon content

## Abstract

Tantalum carbide (TaC) is a highly refractory material with a melting point of 4153 K, making it attractive for applications requiring excellent hardness and thermal stability. In this study, we investigated the carburization behavior of high-purity tantalum metal powder synthesized by magnesium thermal reduction of Ta_2_O_5_, using activated carbon and graphite as carbon sources under high vacuum. Carburization was conducted at 1100–1400 °C for durations of 5–20 h. Carbon contents were analyzed via combustion analysis, and activation energies were calculated based on Arrhenius plots. The results showed that the activated carbon significantly enhanced carbon uptake compared to graphite due to its higher porosity and surface reactivity. The formation and transformation of carbide phases were confirmed via X-ray diffraction, revealing a progression from Ta to Ta_2_C and eventually to single-phase TaC with increasing carbon content. Scanning electron microscopy (SEM) analysis showed that fine particles formed on the surface as carbon content increased, indicating local nucleation of TaC. Although the theoretical carbon content of stoichiometric TaC (6.22 wt.%) was not fully achieved, the near-theoretical lattice parameter (4.4547 Å) was approached. These findings suggest that activated carbon can serve as an effective carburizing agent for the synthesis of TaC under vacuum conditions.

## 1. Introduction

Tantalum carbide (TaC) is a special material with an ultra-high melting point of 4153 K, featuring excellent hardness and thermal resistance [1,2,3]. Owing to its outstanding mechanical properties, TaC is widely recognized as a key material in the defense and electronics industries, including applications such as jet engines, missiles, and capacitors [4,5,6,7]. One of the most remarkable characteristics of TaC is its role as an additive [8,9]. When a small amount of TaC is added during the manufacture of cemented carbides, it effectively suppresses the grain growth of WC and TiC while significantly improving oxidation resistance [10]. Consequently, the demand for TaC powder continues to increase across various industries, especially in the cutting tool industry. Therefore, continuous research and development of TaC powder production technology is essential to strengthen industrial competitiveness and foster technological innovation. Uniform particle size is critical for consistent product quality, and high-purity TaC powder with low oxygen content significantly contributes to enhanced mechanical properties [11].

In general, the TaC synthesis methods can be classified into gas carburization and solid-state carburization. Among solid-state methods, self-propagating high-temperature synthesis (SHS) has attracted attention as an efficient approach [12,13,14]. This method involves mixing tantalum metal powder and graphite powder in a precisely calculated ratio, followed by compaction and ignition. The main feature of SHS is that it utilizes the exothermic reaction of the reactants to sustain the reaction without an external energy supply. Although the SHS offers high energy efficiency and process simplicity, enabling economical production of TaC powder. However, the method is limited by the difficulty of precisely controlling carbon concentration and forming a uniform carburized layer, which may affect product consistency.

Gas-phase carburization, on the other hand, provides better control over phase formation and stoichiometry by reacting tantalum or its oxides with hydrocarbon gases such as methane, propane, butane, or hexane at elevated temperatures [15,16,17]. Despite its advantages, this method is limited by the need for high-purity precursors, specialized gas handling systems, and significant capital costs. For example, methane, though effective, is expensive, and common hydrocarbons like propane and hexane often require complex purification [18].

In our previous work, we have successfully synthesized the TaC using purified hexane; this approach required additional purification and vaporization steps [19]. However, the necessity of vaporization and purification limits its scalability and economic viability for large-scale or industrial applications.

Therefore, in this study, we used activated carbon, which is considerably less expensive and easier to handle than gases (methane or hexane), to synthesize TaC. While previous studies have explored carburization of mild steel using activated carbon, there is still a lack of research on the carburization of tantalum using this method [20,21]. Activated carbon releases a range of gases upon heating, including CO, CO_2_, CH_4_, and various light hydrocarbons below 600 °C, due to the decomposition of oxygen-containing functional groups and residual organics [22]. At higher temperatures (above ~1000 °C), especially under vacuum or inert conditions, minor amounts of carbon vapor species such as diatomic (C_2_) and triatomic carbon (C_3_) may evolve. Graphite powder (GP), in contrast, exhibits a more stable thermal profile and lacks volatile functional groups, releasing such carbon species only under extreme conditions (typically >2000 °C in vacuum) [23]. Accordingly, we attempted to synthesize TaC by exposing tantalum powder to the gaseous species evolved from both activated carbon and graphite—two economical and practical solid carbon sources for solid–gas phase carburization.

This approach addresses critical challenges in existing methods by:

1. Eliminating the need for high-purity gases or vaporization steps;
2. Allowing precise exposure of tantalum to in situ generated gaseous carbon;
3. Proposing a scalable and economical route for producing high-purity TaC.

Furthermore, graphite, known for its thermal stability and minimal volatility, serves as a comparative control to examine the impact of gas composition on carburization behavior.

By systematically evaluating the thermal decomposition behavior of AC and GP and correlating it with TaC formation, this work provides both scientific insight into carbon species transport mechanisms and a technological foundation for scalable solid–gas phase synthesis of metal carbides.

## 2. Experimental Methods

The tantalum metal powder used in this study was prepared by magnesium thermal reduction of tantalum pentoxide (Ta_2_O_5_), as developed in our previous work. The resulting powder was of high purity, containing 0.7 wt.% oxygen, 0.01 wt.% nitrogen, and 0.005 wt.% residual magnesium. Activated carbon (AC, 99% purity, product no. NIST2451, Sigma-Aldrich, St. Louis, MO, USA) and graphite powder (GP, 99.9% purity, CAS No. 7782-42-5, Junsei Chemical, Tokyo, Japan) were used as carburizing agents. Activated carbon is made from carbon-rich materials such as wood, coconut shells, coal, etc. These raw materials are converted into char (carbon-rich solid) by the pyrolysis process (400–900 °C in the absence or low levels of oxygen). The activation is performed by either physical (heating the char in the presence of oxidizing gases, CO_2_) or chemical methods (infusing KOH, ZnCl_2_ before carbonization). This activation resulted in pore development, which provides higher levels (depending on the pore size) of surface area and adsorption and provides pathways for diffusion.

Approximately 10 g of tantalum powder was mixed with 2 g of the carburizing agent and loaded into a graphite crucible. The crucible was then placed in a vacuum furnace capable of maintaining a high vacuum (~10^−5^ Torr).

Carburization was conducted at four different temperatures, starting from 1100, 1200, 1300, and 1400 °C, for 5, 10, and 20 h, respectively. The heating rate was set to 10 °C/min. During the reaction, graphite and activated carbon vaporized at high temperature, generating carbon-containing gases that diffused into the tantalum powder surface and induced carburization. After heat treatment, the furnace was cooled under vacuum and the specimens were collected.

**Figure 1:**
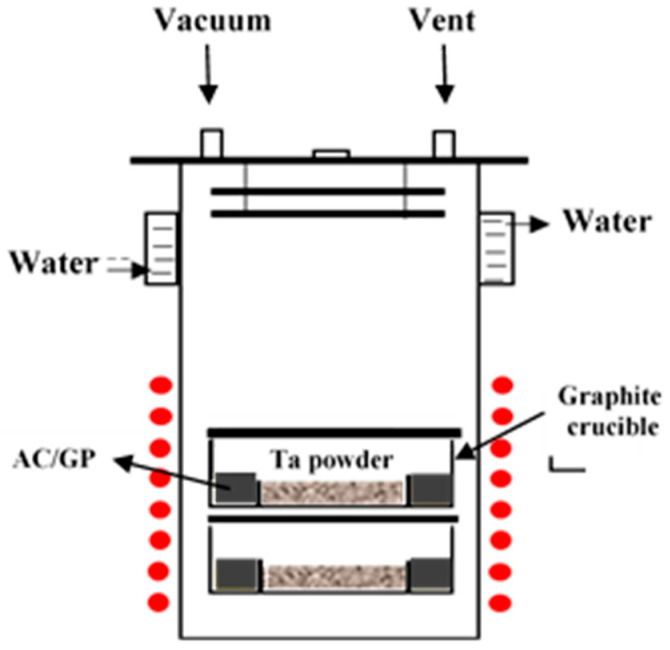
The schematic shows the synthesis process of TaC powder by activated carbon or graphite (red circles: heat).

Carbon content in the carburized tantalum powders was quantitatively analyzed using a combustion-type carbon analyzer (Carbon/Sulfur Determinator, LECO CS-200, LECO, St. Joseph, MI, USA). Based on the obtained carbon concentration data, activation energy was calculated using the Arrhenius equation. X-ray diffraction analysis (Rigaku D/Max-2500, Rigaku, Tokyo, Japan, Cu K_α_, λ = 1.5406 Å) was employed to evaluate changes in the lattice parameter and phase formation as a function of carbon content. Surface morphology and microstructure were observed using a scanning electron microscope (SEM; Hitachi S-4800, Hitachi, Tokyo, Japan), allowing us to analyze the effect of carburization on particle shape and surface state (Figure 1).

## 3. Results and Discussion

**Figure 2:**
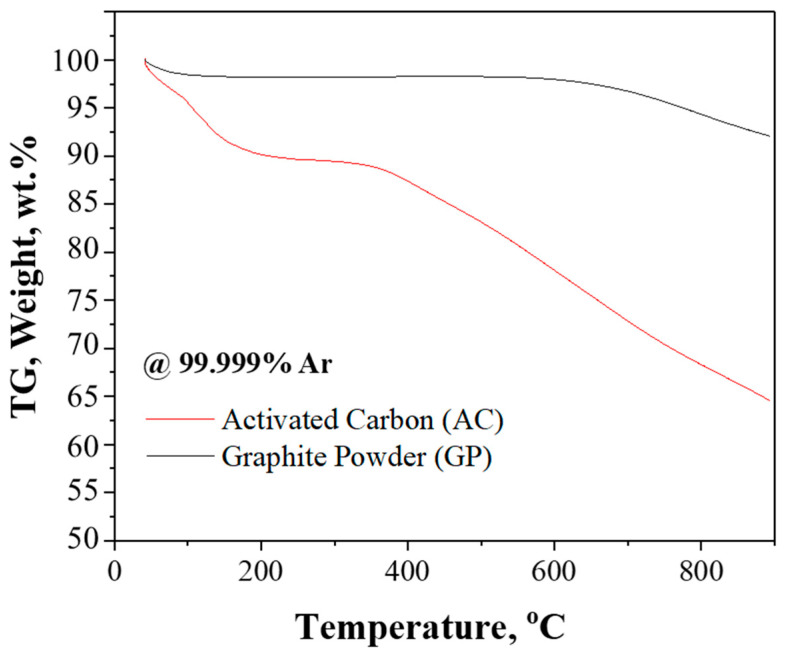
Thermogravimetric analysis (TGA) of activated carbon (AC) and graphite powder (GP) under a 99.999% Ar atmosphere with a heating rate of 10 °C/min up to 900 °C.

To evaluate the thermal behavior of the carburizing agents—graphite and activated carbon—thermogravimetric analysis (TGA) was performed. Approximately 40 mg of each carburizing agent was used for the TGA tests, which were carried out up to approximately 900 °C under a high-purity argon atmosphere (99.999%) at a heating rate of 10 °C/min, and the weight loss characteristics of each carburizing agent were compared. As shown in Figure 2, activated carbon began to show gradual weight loss from around 100 °C and recorded a total mass loss of approximately 17 mg above 800 °C. This behavior suggests the presence of volatile components or carbon-based gases that evolved at relatively low temperatures due to the amorphous and porous structure of activated carbon. In contrast, graphite exhibited minimal weight loss, only above 700 °C, and showed overall stable thermal behavior.

These results clearly demonstrate the differences in carbon volatilization behavior between the two carburizing agents. Under identical heat treatment conditions, activated carbon generated a greater amount of carbon-based gases than graphite, indicating that it could more actively promote the carburization reaction. Therefore, in subsequent carburization experiments, the impact of this carbon generation characteristic on the extent of carburization and reaction rate in tantalum powders was analyzed and compared.

**Figure 3:**
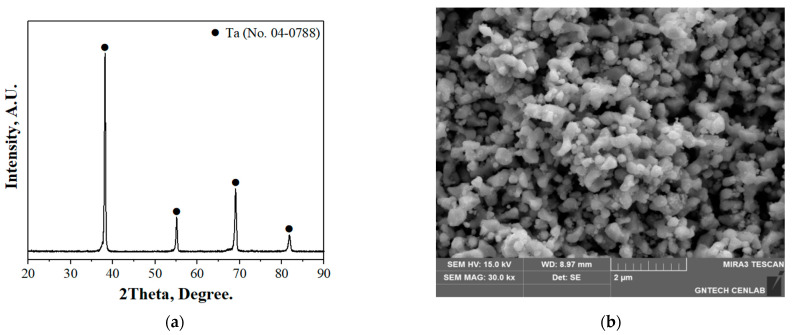
(**a**) X-ray diffraction pattern and (**b**) scanning electron microscopy image of tantalum metal powder extracted by magnesium reduction of Ta_2_O_5_.

Figure 3 presents the results of X-ray diffraction (XRD) and scanning electron microscopy (SEM) for the tantalum metal powder synthesized by magnesium reduction used in this study [24]. The XRD pattern confirmed the presence of a single-phase Ta structure. SEM analysis revealed that the reduced powder generally exhibited irregular shapes composed of agglomerated particles ranging in size from several tens of nanometers to several micrometers. Upon closer observation, individual particles were found to exhibit satellite-like morphology, with fine particles attached around a coarse core. This morphology is attributed to condensation or agglomeration of vapor-phase reduced products during the high-temperature reduction process. Such a polydisperse structure, composed of both coarse and fine particles, may influence carbon diffusion pathways during the carburization process. In particular, the fine particles with larger surface area could provide higher carbon absorption capacity during the early stages of the reaction.

**Figure 4:**
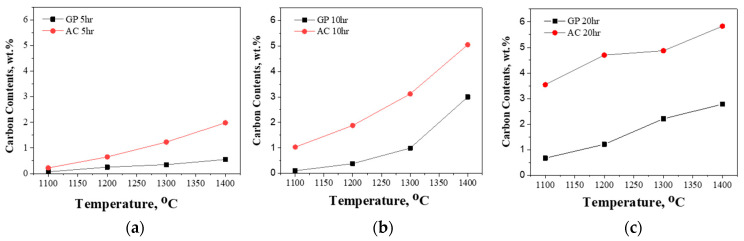
Carbon content (wt.%) of carburized tantalum powders as a function of carburization temperature under various time intervals: (**a**) 5 h, (**b**) 10 h, and (**c**) 20 h.

Figure 4 shows the carbon content of tantalum powders carburized under various conditions, including different times, temperatures, and types of carburizing agents. Each graph corresponds to carburization durations of 5, 10, and 20 h, with temperatures ranging from 1100 to 1400 °C. As the carburization time increased, the carbon content also showed a tendency to increase. This trend was particularly pronounced when activated carbon was used as the carburizing agent. This can be attributed to the fact that activated carbon generates a larger amount of carbon-containing gas than graphite, thereby promoting surface reactions and diffusion. In addition, extended carburization time allowed more internal diffusion of carbon, resulting in a higher average carbon content. Similarly, increasing the carburization temperature also led to a significant rise in carbon content. In particular, when activated carbon was used at 1400 °C for 20 h, a carbon content of 5.83 wt.% was achieved. This result is due to enhanced carbon diffusion coefficients at high temperatures and increased activity of gas-phase reactions. In contrast, graphite consistently exhibited lower carbon content than activated carbon, even under high-temperature conditions, indicating inherent limitations in carburization using graphite. These results highlight that under identical carburization conditions, activated carbon results in higher carbon uptake than graphite. This observation aligns qualitatively with the TGA results shown in Figure 2, further supporting the higher reactivity and volatilization characteristics of activated carbon.

**Figure 5:**
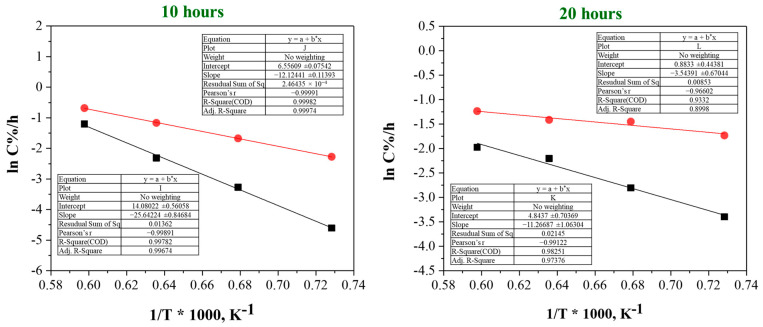
Arrhenius plots derived from the carburization of tantalum metal powders using graphite (black line) and activated carbon (red line) as carbon sources at carburization durations of 10 h and 20 h. The y-axis represents the natural logarithm of the carbon diffusion coefficient (ln K^−1^).

Figure 5 presents the activation energy results calculated using the Arrhenius equation, based on the carbon content data obtained after carburization heat treatment. To analyze the kinetics of the carburization reaction, we assumed a simplified first-order reaction model where the carburization rate is proportional to the amount of uncarburized tantalum surface available for reaction. Accordingly, the carburization rate constant, k, was determined by dividing the carburized carbon content by the carburizing time, representing an average reaction rate over the heat treatment period.

The temperature dependence of k was then evaluated using the Arrhenius equation, which describes the reaction rate constant as the product of a pre-exponential factor and the exponential of negative activation energy divided by the product of the gas constant and absolute temperature. By plotting the natural logarithm of k against the inverse of the temperature, the activation energy was obtained from the slope of the linear fit.

The results reveal distinct differences in activation energy depending on the carburization time and carbon source. For the 10 h treatment, the activation energy was calculated as 212 kJ/mole for graphite and 101 kJ/mole for activated carbon. At 20 h, the values decreased to 93 and 29 kJ/mole, respectively. This decreasing trend suggests a change in the rate-limiting step over time: initial stages are likely dominated by surface reaction and nucleation processes, while prolonged carburization leads to diffusion-controlled mechanisms within the tantalum lattice. The lower activation energy for activated carbon, especially at early stages, can be attributed to its amorphous and porous nature, which facilitates rapid carbon release and surface reaction compared to crystalline graphite.

While this simplified kinetic model provides a useful approximation to evaluate activation energy, further detailed kinetic studies incorporating diffusion and phase transformation effects are warranted to fully elucidate the carburization mechanism.

**Figure 6:**
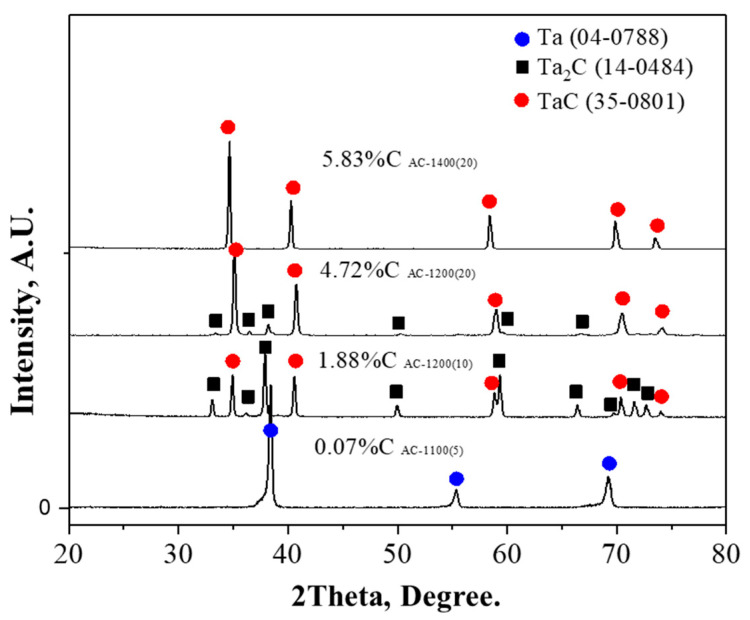
X-ray diffraction (XRD) patterns of carburized tantalum powders with varying carbon contents.

Figure 6 shows the X-ray diffraction (XRD) results corresponding to different carbon contents in tantalum powders obtained through representative carburization conditions. The XRD analysis revealed a clear variation in the composition of carbide phases as the carbon content increased. At a carbon content of approximately 0.07 wt.%, only pure Ta phase was detected, indicating that the carburization was in its early stages and that insufficient carbon had penetrated the lattice or formed carbides. When the carbon content increased to 1.88 wt.%, both Ta_2_C and TaC phases appeared, indicating a two-phase region where sufficient carbon had diffused into the metal, leading to the formation of multiple carbide species. With a further increase in carbon content to 4.72 wt.%, the peaks corresponding to the Ta_2_C phase diminished or nearly disappeared in the XRD pattern, while TaC emerged as the dominant phase. This suggests a progression toward a more thermodynamically stable TaC composition as the carburization advanced. Finally, at a carbon content of 5.83 wt.%, only the TaC phase was observed, confirming that the reaction had proceeded sufficiently to reach a nearly saturated carburized state. These results indicate that as both carburization time and temperature increased, carbon continued to diffuse and react with tantalum, ultimately resulting in the formation of a single-phase TaC structure.

**Figure 7:**
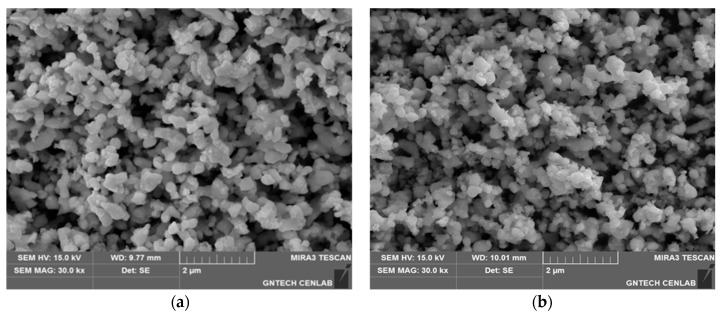
Scanning electron microscope (SEM) images of carburized tantalum powders with different carbon contents: (**a**) 0.07 wt.% carbon and (**b**) 5.83 wt.% carbon.

Figure 7 compares the microstructures of carburized tantalum powders observed by SEM, focusing on specimens with carbon contents of 0.07 wt.% (left) and 5.83 wt.% (right). Both samples show aggregated polyhedral particles, but significant differences emerge at higher carbon levels. In the 5.83 wt.% sample, numerous ultrafine particles are present on and between the larger particles, indicating additional nucleation sites and enhanced surface activity. These morphological changes suggest that higher carbon concentrations promote heterogeneous nucleation of TaC on existing particle surfaces, rather than simple interstitial diffusion. The formation of nanoscale secondary particles likely reflects local supersaturation of carbon, which drives phase separation and carbide precipitation. This supports a non-uniform, surface-initiated carburization mechanism at elevated carbon levels.

Furthermore, the increased surface roughness and fine particle generation may indicate surface reconstruction phenomena, possibly due to rapid diffusion of carbon at grain boundaries or surface defects. This correlates with the enhanced reactivity and kinetic pathway observed under high-temperature solid–gas reactions.

To evaluate the changes in the lattice parameter of tantalum powders as a function of carbon content, X-ray diffraction analysis was used (Figure 6). The lattice parameter can be determined based on the diffraction angles obtained from X-ray diffraction analysis and the wavelength of the incident X-rays. Diffraction occurs at specific crystallographic planes within the crystal, and the measured diffraction angles are converted into interplanar spacings using Bragg’s law [25,26]. These spacings are mathematically related to the lattice parameter depending on the crystal structure and the Miller indices of the diffracting planes. The indexed diffraction peaks in the XRD pattern can be utilized to calculate the lattice parameter of the crystal lattice.

**Figure 8:**
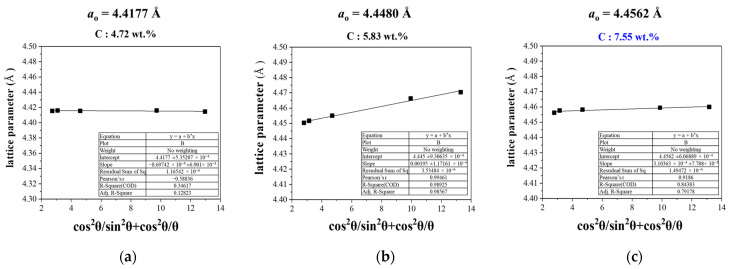
Result of calculation of lattice parameter according to carbon contents: (**a**) 4.72 wt.% carbon, (**b**) 5.83 wt.% carbon, and (**c**) 7.55 wt.%.

To improve the accuracy of the lattice parameter determination, correction functions such as the Nelson–Riley function are applied to compensate for systematic errors arising during the measurement process [27,28,29]. The results in Figure 8 show a gradual increase in the lattice parameter with increasing carbon content after carburization. Specifically, the sample with a carbon content of 4.72 wt.% exhibited a lattice parameter of 4.4177 Å, while the 5.83 wt.% sample showed a value of 4.4480 Å. A reference sample with 7.55 wt.% carbon content exhibited a lattice parameter of 4.4562 Å. Compared to the theoretical lattice parameter of TaC (4.4547 Å), these results indicate that higher carbon contents correspond to values approaching the theoretical parameter.

This increase in lattice parameter is interpreted as the result of carbon atoms increasingly diffusing and being incorporated into the interstitial sites of the tantalum lattice during the carburization process, leading to expansion of the crystal structure. The 4.4480 Å value at 5.83 wt.% carbon content closely matches the theoretical value, suggesting that the TaC phase is well developed and structurally stabilized. In contrast, the 4.72 wt.% sample still showed signs of coexisting Ta_2_C phase, which may have resulted in the slightly lower lattice parameter.

**Figure 9:**
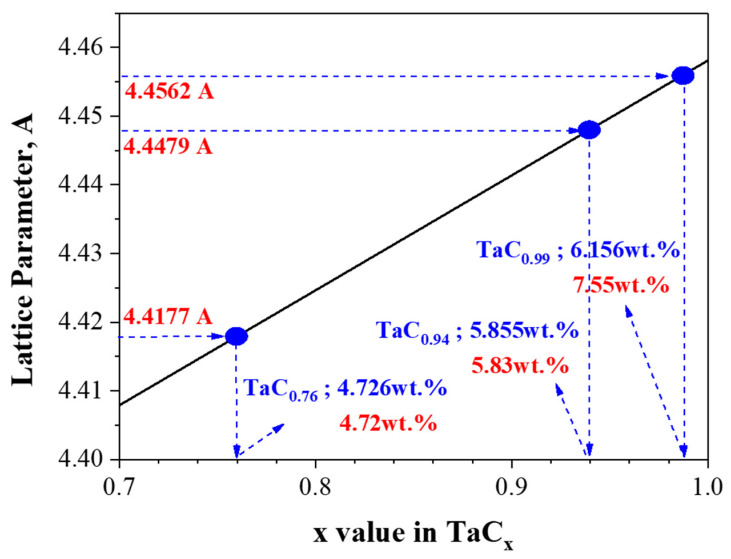
Variation in the lattice parameter of TaC_x_ as a function of carbon content. Experimental values (red) were obtained from measured compositions (4.72, 5.83, and 7.55 wt.%), while theoretical values (blue) are shown for reference.

Figure 9 shows the correlation between the carbon content (in wt.%) and the lattice parameter of the carburized tantalum carbide (TaC_x_) samples. The data clearly show that as the carbon content increases, the lattice parameter also increases. This trend is consistent with the known behavior of nonstoichiometric TaC phases, where carbon deficiency leads to lattice contraction, and increasing carbon content results in lattice expansion.

At a carbon content of approximately 4.72 wt.%, the lattice parameter is 4.4177 Å, corresponding to a substoichiometric composition near TaC_0.75_. As the carbon content increases to around 7.55 wt.%, the lattice parameter approaches 4.4562 Å, which aligns with the stoichiometric TaC_1.0_. These results suggest that the lattice parameter can serve as an indirect but reliable indicator of carbon content and thus the stoichiometry of the carbide phase.

To quantitatively estimate the carbon-to-tantalum atomic ratio (C/Ta), we referred to the empirical relationship reported by B. Mehdikhan et al. in “Effect of Milling and Sintering Temperature of TaC–TaB_2_ Composite on Lattice Parameter and C/Ta Ratio” [30]. According to their study, the C/Ta ratio can be expressed as a function of the lattice parameter *a*_0_ using the following equation:

C/Ta = −25.641 + 5.9757 *a*_0_

Using this equation, the C/Ta ratios for our samples were calculated based on the measured lattice parameters, providing further insight into the stoichiometry of the formed TaC_x_ phases.

Although the carbon content in our study does not reach the theoretical stoichiometry of TaC, the proposed carburization method is capable of forming a range of substoichiometric phases. Moreover, the clear correlation between lattice parameter and carbon content provides a useful approach for estimating the composition of the obtained carbides and supports further optimization of the synthesis conditions.

Therefore, the lattice parameter measurements serve as a crystallographic indicator of the progression of the carburization reaction. As carbon content increases sufficiently, a pure TaC crystal phase is formed, and the lattice structure approaches the theoretical equilibrium state. Accordingly, even under identical conditions, the characteristics of the carbon source and the carburization time have a decisive impact on the reaction mechanism and activation energy. Activated carbon is evaluated as an efficient carbon source that can promote rapid carburization in a short time, while graphite tends to enable more stable carburization reactions over extended durations. Therefore, when designing carburization conditions, the selection of carbon source and heat treatment time are critical factors for optimizing process efficiency and controlling the final material properties.

## 4. Conclusions

1. Even under the most intensive carburization condition (1400 °C, 20 h), the carbon content of the carburized tantalum powder reached 5.83 wt.%, which did not attain the theoretical composition of TaC (6.22 wt.%). This result indicates that the saturation limit of the carburization reaction was not fully reached and suggests that multiple diffusion and reaction conditions influenced the outcome.

2. The carburization process involves vaporized carbon gases diffusing upon the metal surface at high temperatures and subsequently dissolving into the lattice to form carbides. However, under the high-vacuum conditions used in this study, the partial pressure of carbon was likely relatively low, which may have resulted in an insufficient concentration of carbonaceous species at the tantalum surface.

3. Moreover, carbon diffusion and the formation of tantalum carbide require overcoming an energy barrier. If the carburization temperature and duration are not sufficient, complete carbide formation becomes difficult. In fact, although the measured lattice parameter approached the theoretical value for TaC (4.4547 Å), the carbon content did not reach the theoretical stoichiometry. This implies that the transformation progressed structurally, but saturation was not achieved in terms of carbon composition.

4. Therefore, to achieve complete formation of the TaC phase, process conditions must be optimized by either extending the heat treatment duration or increasing the carbon concentration and partial pressure. Future research may consider the introduction of external carbon gases or the use of elevated pressures or more reactive atmospheres to enhance the carburization process.

## Data Availability

The original contributions presented in this study are included in the article. Further inquiries can be directed to the corresponding author.
